# Virtual reality simulation training in laparoscopic surgery – does it really matter, what simulator to use? Results of a cross-sectional study

**DOI:** 10.1186/s12909-024-05574-0

**Published:** 2024-05-28

**Authors:** Moritz B. Sparn, Hugo Teixeira, Dimitrios Chatziisaak, Bruno Schmied, Dieter Hahnloser, Stephan Bischofberger

**Affiliations:** 1 Department of Surgery, Kantonsspital St. Gallen, CH-9007 St. Gallen, Switzerland; 2 Department of Surgery, Centre Hospitalier Universitaire Vaudois, CH-1011 Lausanne, Switzerland

**Keywords:** Virtual Reality Training, Simulation, Laparoscopic Training, Surgical Training, Surgical Education

## Abstract

**Background:**

Virtual reality simulation training plays a crucial role in modern surgical training, as it facilitates trainees to carry out surgical procedures or parts of it without the need for training “on the patient”. However, there are no data comparing different commercially available high-end virtual reality simulators.

**Methods:**

Trainees of an international gastrointestinal surgery workshop practiced in different sequences on LaparoS® (VirtaMed), LapSim® (Surgical Science) and LapMentor III® (Simbionix) eight comparable exercises, training the same basic laparoscopic skills. Simulator based metrics were compared between an entrance and exit examination.

**Results:**

All trainees significantly improved their basic laparoscopic skills performance, regardless of the sequence in which they used the three simulators. Median path length was initially 830 cm and 463 cm on the exit examination (*p* < 0.001), median time taken improved from 305 to 167 s (*p* < 0.001).

**Conclusions:**

All Simulators trained efficiently the same basic surgery skills, regardless of the sequence or simulator used. Virtual reality simulation training, regardless of the simulator used, should be incorporated in all surgical training programs. To enhance comparability across different types of simulators, standardized outcome metrics should be implemented.

**Supplementary Information:**

The online version contains supplementary material available at 10.1186/s12909-024-05574-0.

## Background

Minimally invasive surgery (MIS) is considered standard of care in many surgical procedures nowadays and laparoscopic techniques have revolutionized surgery in numerous fields [1]. The rise of robotic-assisted techniques is currently accelerating the growth of minimally invasive surgery. Acquiring the skills needed to perform laparoscopic procedures safely can be challenging. Limited working hours, increased patient safety requirements, as well as increasing time pressures in the operating room, make it difficult to learn and teach MIS “on the job,” as has been Halsted’s apprentice-tutor model for nearly a century.

The efficacy of virtual reality simulation training (VRST) has been shown in multiple studies among other things reducing error rates and improving working speed [2–5]. VRST is important for early technical skills acquisition in laparoscopic surgery [6]. Several randomized controlled trials highlighted their growing importance as a safe, ethical, and comparable way to train basic surgical skills [7–9].

Currently, several virtual reality simulators (VRS) are available from various companies. These offer several training options and curricula including basic tasks (e.g., camera guidance, bimanual working, eye-hand coordination), advanced skills (e.g., suturing, knot tying) and complete surgical procedures (e.g., laparoscopic cholecystectomy, appendectomy). However, there is no generally accepted standard for VRST, although they train similar basic and procedural skills. So far, however, there is no evidence of a different training effect of the various virtual reality simulators.

The aim of this study was to evaluate the training of similar basic surgical skills on three different VRS and to determine whether the participants' performance was influenced by the training sequence and the simulators used.

## Methods

The dataset of the presented study was obtained during the 38th annual Davos Course in 2021, an international surgical training course in Davos, Switzerland (www.davoscourse.ch). The six-day course offers a blended learning experience with theoretical parts and a strong emphasis on hands-on training (open, laparoscopic & robotic).

Three different virtual reality simulators (VRS) were used: LapSim® (Surgical Science Sweden AB, Gothenburg, Sweden), LAP Mentor III® (Simbionix, Tel Aviv, Israel), and LaparoS® (VirtaMed AG, Zurich, Switzerland). The companies provided the simulators without any financial benefit and without sponsoring of the Davos course. Employees from the companies were present during the training and experienced surgeons acted as instructors. The study protocol was developed independently of the simulator companies but was approved by them before the start of the study.

We obtained written informed consent from all study participants. Data collection was completely anonymized. Swiss Ethics Committee "Swissethics.ch" grants a general waiver for the use of purely anonymized data and an application with approval is not requested (Swiss Federal Act on “Research involving Human Beings 810.30”). All methods were carried out in accordance with relevant guidelines and regulations.

**Fig. 1:**
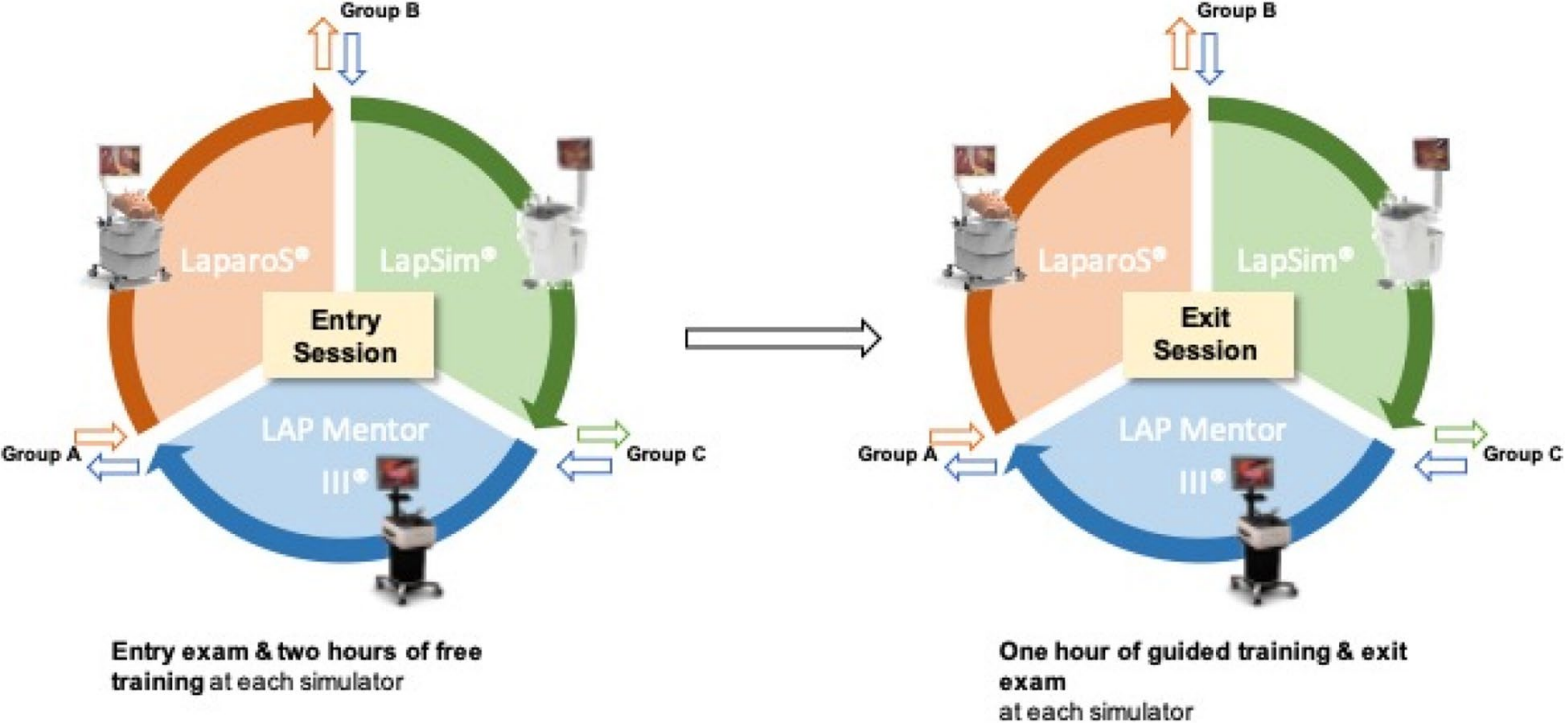
Schedule of all participants during their virtual reality simulation training

Study participants were randomly assigned into three groups. All participants trained in time slots of two hours each on three consecutive days with one of the available three different VR simulators. Each participant started their timeslot with an entry exam of three exercises, followed by two hours of free training. Participants then moved to the next simulator, where they repeated that sequence (entry exam, two hours of training). After having trained on each of the three simulators, participants then returned to all three simulators in the same sequence for one hour of training and repeated the initial assessment as an exit exam (Fig. 1). The exercises performed on each simulator are listed in Appendix 1 and aimed to train the following skills: bimanual coordination, eye-hand-coordination, safe application of technical devices (e.g. clips) and tissue dissection. This manuscript was written according to the STROCCS 2021 guideline [10].

For each participant, the following simulator metrics were collected: the total path length of all instruments used, and time taken to complete the exercises. Continuous data are given as median (IQR) or mean (SD) as appropriate.

### Statistical analyses

Participants’ baseline data by group were compared using the chi-square test or Kruskal–Wallis Rank Sum test, as appropriate. Entry and exit examinations were compared overall and groupwise using Welch’s t-test for total path length as well as total time used [11]. The association of total time used and total instruments’ path length for all entry and all exit examinations, grouped by simulators, was evaluated using scatterplots with locally estimated scatterplot smoothers with a confidence interval of 95% (LOESS smoothers) [12]. A two-sided *p*-Value of < 0.05 was considered statistically significant. All statistical analyses were performed using R studio, version 2022.12.0 (www.r-project.org, Vienna, Austria).

## Results

Eighteen participants took part in the study. There were no significant differences in surgical and training experience among the three groups (Table 1).

**Table 1:** Baseline data of participants by groups

**First Simulator**		**SIM (*n* = 6)**	**SS (*n* = 6)**	**VM (*n* = 6)**	***p*-Value**
Gender	Female	4	5	3	0.472
	Male	2	1	3	
Dexterity	Right	6	6	6	
Years of surgical training	1–3	5	6	5	0.119
Lap. Cholecystectomies performed	≤ 10	2	3	2	0.204
	11–25	4	3	4	
Lap. Appendectomies performed	≤ 10	3	5	6	0.423
	11–25	3	1	0	
Lap. Sigmoid Resections performed	≤ 10	2	5	6	0.204
	11–15	4	1	0	

*VM* VirtaMed LaparoS®, *SIM* Simbionix LAP Mentor III®, *SS* Surgical Science LapSIM®

**Fig. 2:**
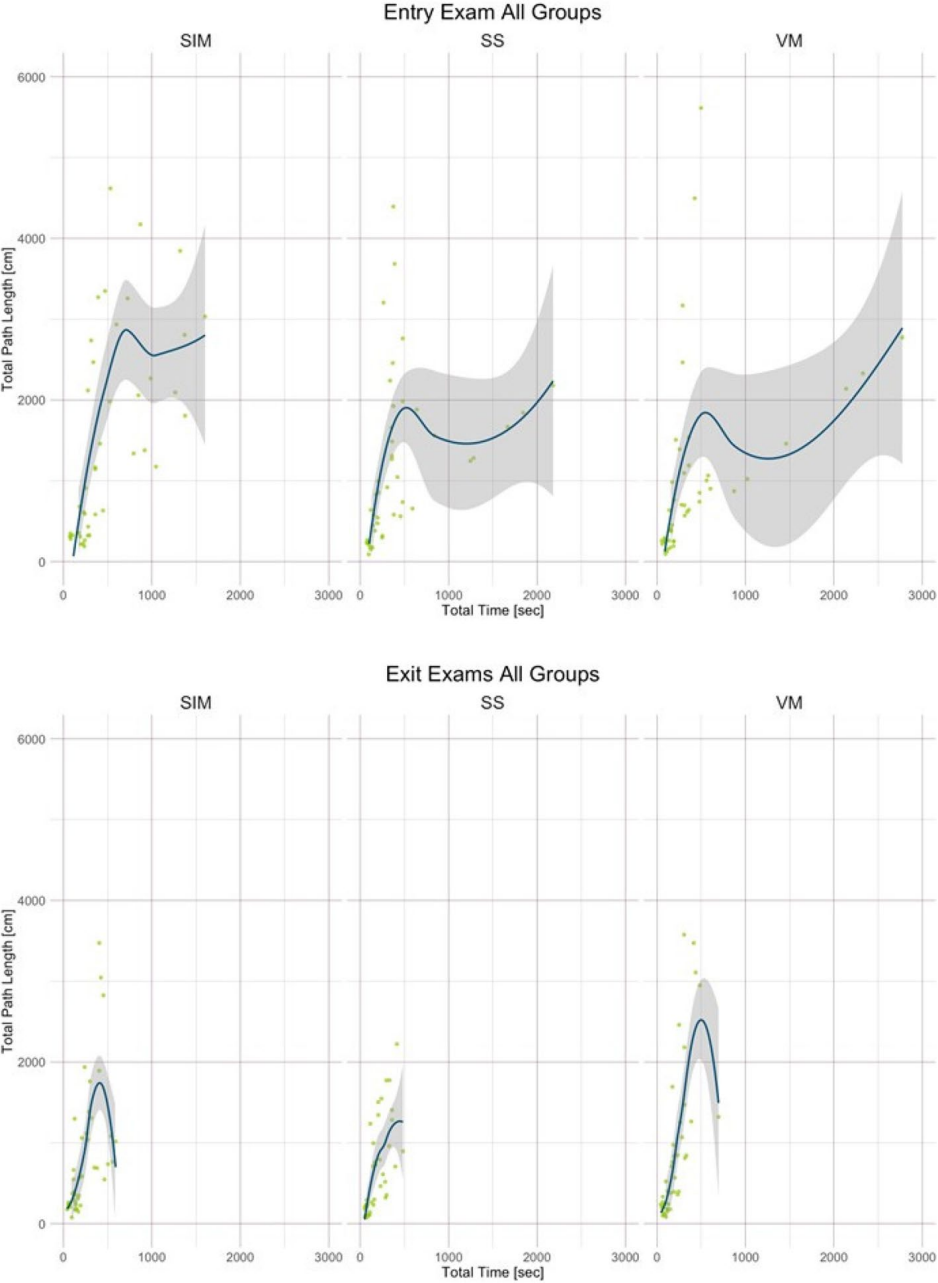
Scatterplots of total path length and total time on Entry and Exit Exams with LOESS smoothers, grouped by Simulators, SIM = Simbionix LapMentor III®, SS = Surgical Science LapSim®, VM = VirtaMed LaparoS®

In total, participants performed 135 entry examinations and 127 exit examinations on the three different simulators. The median total path length on entry examination was 830, 25 cm (IQR 322.60–1823.57) and 462.96 cm (IQR 224.96–1063.33) on exit examination (*p* < 0.001). Median time taken was 305.40 s (IQR 168.70–481.42) and 166.80 s (IQR 98.92–291.56), respectively (*p* < 0.001). The improvements are visualized as Scatterplots in Fig. 2. Forty-four (32.6%) of all entry examinations were performed on the LAP Mentor III (SIM) simulator, 45 (33.3%) on the LapSim (SS) simulator and 46 (34.1%) on the LaparoS (VM) simulator. Forty-four (34.6%) exit examinations were performed on SIM, 40 (31.5%) on SS, and 43 (33.9%) on VM. In all groups, there was a significant reduction in time taken to complete the exit exercises compared to the entry examinations. Path lengths were significantly reduced in participants using LAP Mentor III and LapSim, but not in participants starting on the LaparoS. Table 2 shows the different exercises. The exercises are described in detail in Appendix 1 and in several publications [7, 13, 14]. Table 3 shows time and path lengths of the three different groups. Table 4 shows total time and total path length at entry and exit examinations by the different exercises conducted. There was a statistically significant improvement of time needed in all exercises except PVT1, a knot-tying exercise. Path lengths were significantly lower in the following exercises: Gallbladder resection, Lap Chole Task 2, Lap Chole Task 3, Task 9, and PVT2 (see Appendix 1, Table 4).

**Table 2:** Number of exercises performed on the three simulators

Exercise Name (Company)	Entry Examination *n* = 135	Exit Examinations *n* = 127
Gallbladder Resection (VM)	16	11
Grasping and bimanual coordination (VM)	16	18
Vascular Injury repair (VM)	13	12
LCHE Task 2: Clipping and Cutting with Two Hands (SIM)	18	18
LCHE Task 3: Dissection—Critical View of Safety’ (SIM)	18	18
Task 9: Translocation of Objects (SIM)	18	17
PV/T1: Suturing (SS)	18	15
PV/T2: Lifting & grasping (SS)	18	18

*VM* VirtaMed LaparoS®, *SIM* Simbionix LAP Mentor III®, *SS* Surgical Science LapSIM®, *LCHE* Laparoscopic Cholecystectomy

**Table 3:** Differences of the mean were analyzed by Welch’s t-test

First Simulator	Path Length Entry Examinations [cm] (SD)	Path Length Exit Examinations	*p*-Value	Total time Entry Examinations [seconds] (SD)	Total Time Exit Examinations [seconds] (SD)	*p*-Value
VirtaMed LaparoS	1040.32 (1140.32)	878.49 (959.96)	0.47	449.42 (592.43)	201.34 (137.85)	0.008
Simbionix LAP Mentor III	1478.12 (1263.97)	798.02 (804.35)	0.003	502.12 (410.09)	229.2908 (154.86)	< 0.001
Surgical Science LapSIM	1170.06 (1004.48)	654.94 (574.91)	0.004	502.12 (475.79)	229.29 (118.57)	< 0.001

*VM* VirtaMed LaparoS®, *SIM* Simbionix LAP Mentor III®, *SS* Surgical Science LapSIM®

*p* < 0.05 is considered statistically significant

**Table 4:** Total path lengths and total time used at entry and exit examination by exercises

Exercise Name (Simulator)	Mean Path Length on Entry examination (SD) [cm]	Mean Path Length on Exit examination (SD) [cm]	*p*-Value	Mean Time on Entry examination (SD) [s]	Mean Time Exit (SD) [s]	*p*-Value
Gallbladder resection (VM)	1882.96 (741.2)	722.38 (284.77)	< 0.001	1508.35 (594.39)	335.69 (126.87)	< 0.001
Grasping Bimanual (VM)	1674.40 (1048.95)	1124.52 (648.29)	0.082	400.69 (222.89)	227.14 (112.19)	0.0102
Vascular Injury (VM)	425.58 (145.34)	325.86 (157.85)	0.11	206.63 (68.69)	140.83 (59.36)	0.017
Lap Chole Task 2 (SIM)	200.40 (69.92)	142.82 (58.21)	0.011	173.37 (66.73)	110.60 (36.15)	0.001
Lap Chole Task 3 (SIM)	1190.14 (742.41)	457.34 (340.57)	< 0.001	714.16 (375.50)	282.18 (196.24)	< 0.001
Task 9 (SIM)	3108.51 (1120.32)	2248.82 (836.17)	0.014	361.44 (92.01)	287.92 (108.11)	0.038
PVT1 (SS)	907.54 (372.11)	917.35 (449.43)	0.94	284.12 (82.37)	246.05 (89.74)	0.218
PVT2 (SS)	320.86 (113.99)	233.01 (37.14)	0.005	95.75 (32.13)	61.89 (9.92)	< 0.001

Differences of the mean were analyzed by Welch’s t-test

*VM* VirtaMed LaparoS®, *SIM* Simbionix LAP Mentor III®, *SS* Surgical Science LapSIM®

## Discussion

In this study, we assessed the effect of basic laparoscopic virtual reality simulation training (VRST) on three different commercially available virtual reality simulators. Participants significantly reduced the time needed to complete tasks, and reduced path length of basic skills exercises and simulated cholecystectomy. The overall improvement of path lengths did not reach statistical significance on the LaparoS®, possibly due to the specific complexity of the exercises given. When assessing the single exercises (Table 4), there is a clear improvement of path lengths in the “Gallbladder Resection” exercise and a clear trend in the “Grasping Bimanual” exercise. However, in the “Vascular Injury” exercise, the improvement is not statistically significant. Nevertheless, all participants improved their skills to some extent, regardless the sequence of VRST or simulator used. Simulation in surgical training is considered a well-established technique of honing the skills necessary to perform surgical procedures. To date, multiple studies have shown that simulation training and, more specifically, virtual reality training is superior compared to the traditional surgical apprenticeship-model [6, 7, 9, 15]. Several meta-analyses summarize up to 31 randomized controlled trials evaluating the efficacy of VRST [5, 6, 9, 15]. As one would expect, in most publications, VRST is superior to traditional surgical training and conventional box trainers. There are different modalities in which VRST is held, proficiency based progression (PBP) being considered to be the most effective [7–9]. This particular training method requires a surgical task to be characterized into its individual parts and a benchmarking must be done to clearly define optimal, suboptimal and erroneous performance [16]. Moreover, PBP requires maintenance of a close supervision and repeated formative feedback [17]. All this leads to a relevant amount of infrastructure and personnel in order to meet the requirements of PBP. In this study, we did not evaluate PBP due to limited time, but could demonstrate that similar basic laparoscopic skills can be trained on different VRS available. The shortage of specialists already affects the whole of medicine and in particular the surgical subjects, more so in rural areas than in the cities. This shortage affects the US and Europe alike and different surgical specialties are involved [18–21]. Maintaining surgical training will be a challenge, especially in smaller hospitals that are affected by these bottlenecks. To date, modern technology (e.g. VRS) still has not become an integral part of surgical training. In this study, we show that free training can indeed lead to relevant improvements in basic surgical tasks and in more complex exercises, regardless of the simulator used. The strength in this study is that similar skills were trained on the different simulators, such as bimanual tissue or object handling, safe application of clips and tissue dissection. One major advantage of VRST is that even in with little external guidance and formal structure, VRST provides the unique opportunity to improve one’s individual surgical skills by enabling deliberate practice from the very beginning of a surgical career.

There are several limitations to this study. First, although the simulators are structurally similar and contain similar exercises, the specific metrics cannot be compared. Therefore, we chose to use a “before-after-comparison”, in order to show the effects of VRST itself rather than trying to evaluate the efficacy of specific exercises. This has been done extensively in several other studies and publications. Secondly, we only used total time and total path length of all instruments as surrogate parameters for proficiency/accuracy. It is well known that a task can be performed quickly, but badly executed and meticulously, but in a longer period. The same is true for path length as a surrogate for efficacy of movements. However, both metrics are widely used and enable universal comparability of different simulators and exercises without having to rely on proprietary evaluation algorithms from the manufacturers themselves. Furthermore, implementing and maintaining a mandatory VRST, including structured feedback and expert guidance, involves significant costs that may be prohibitive, especially for smaller, non-academic institutions that train few trainees. A centralized structure with training centers open to surgical trainees from different institutions could be one way of addressing this issue. Additionally, a combined approach to simulation training by the use of VRS such as the ones used in this study and conventional simulators such as box-trainers (e.g. FLS Box) could be a promising way to enhance surgical training.

## Conclusion

This study showed that VRST leads to significant improvement already in short periods of time and with less-than-ideal training modalities, regardless of the sequence in which simulators were used. All VRS trained efficiently the same basic surgery skills, regardless of the sequence or simulator used. This should encourage surgical educators and trainees alike to adopt VRST as an integral part of basic surgical skills training. VRST, regardless of the simulator used, should be incorporated in all surgical training programs. However, standardized and validated outcome metrics should be implemented to reliably measure proficiency and performance of trainees.

### Supplementary Information

Supplementary Material 1.

## Data Availability

The datasets used and analyzed during the current study are available from the corresponding author on reasonable request.

## Abbreviations

MIS: Minimally invasive surgery
VRST: Virtual reality simulation training
VRS: Virtual reality simulators
PBP: Proficiency based progression

## References

[1] Gallagher AG, Ritter EM, Champion H, Higgins G, Fried MP, Moses G, Smith CD, Satava RM (2005). Virtual reality simulation for the operating room. Ann Surg.

[2] Våpenstad C, Hofstad EF, Bø LE, Chmarra MK, Kuhry E, Johnsen G, Mårvik R, Langø T (2013). Limitations of haptic feedback devices on construct validity of the LapSim® virtual reality simulator. Surg Endosc.

[3] Aggarwal R, Warren O, Darzi A (2007). Mental training in surgical education: a randomized controlled trial. Ann Surg.

[4] Aggarwal R, Moorthy K, Darzi A (2004). Laparoscopic skills training and assessment. Br J Surg.

[5] Alaker M, Wynn GR, Arulampalam T (2016). Virtual reality training in laparoscopic surgery: a systematic review & meta-analysis. Int J Surg.

[6] Gurusamy K, Aggarwal R, Palanivelu L, Davidson BR (2008). Systematic review of randomized controlled trials on the effectiveness of virtual reality training for laparoscopic surgery. Br J Surg.

[7] Ahlberg G, Enochsson L, Gallagher AG, Hedman L, Hogman C, McClusky DA, Ramel S, Smith CD, Arvidsson D (2007). Proficiency-based virtual reality training significantly reduces the error rate for residents during their first 10 laparoscopic cholecystectomies. Am J Surg.

[8] Seymour NE, Gallagher AG, Roman SA, O’Brien MK, Bansal VK, Andersen DK, Satava RM (2002). Virtual reality training improves operating room performance. Ann Surg.

[9] Mazzone E, Puliatti S, Amato M, Bunting B, Rocco B, Montorsi F, Mottrie A, Gallagher AG (2021). A systematic review and meta-analysis on the impact of proficiency-based progression simulation training on performance outcomes. Ann Surg.

[10] Mathew G, Agha R, Albrecht J, Goel P, Mukherjee I, Pai P, D’Cruz AK, Nixon IJ, Roberto K, Enam SA, Basu S, Muensterer OJ, Giordano S, Pagano D, Machado-Aranda D, Bradley PJ, Bashashati M, Thoma A, Afifi RY, Johnston M, Challacombe B, Ngu JC-Y, Chalkoo M, Raveendran K, Hoffman JR, Kirshtein B, Lau WY, Thorat MA, Miguel D, Beamish AJ, Roy G, Healy D, Ather HM, Raja SG, Mei Z, Manning TG, Kasivisvanathan V, Rivas JG, Coppola R, Ekser B, Karanth VL, Kadioglu H, Valmasoni M, Noureldin A, STROCSS Group. STROCSS (2021). Strengthening the reporting of cohort, cross-sectional and case-control studies in surgery. Int J Surg Lond Engl..

[11] West RM (2021). Best practice in statistics: use the Welch t-test when testing the difference between two groups. Ann Clin Biochem.

[12] Jacoby WG (2000). Loess: a nonparametric, graphical tool for depicting relationships between variables. Elect Stud.

[13] von Websky MW, Vitz M, Raptis DA, Rosenthal R, Clavien PA, Hahnloser D (2012). Basic laparoscopic training using the Simbionix LAP Mentor: setting the standards in the novice group. J Surg Educ.

[14] Von Websky MW, Raptis DA, Vitz M, Rosenthal R, Clavien PA, Hahnloser D (2013). Access to a simulator is not enough: the benefits of virtual reality training based on peer-group-derived benchmarks—a randomized controlled trial. World J Surg.

[15] Sutherland LM, Middleton PF, Anthony A, Hamdorf J, Cregan P, Scott D, Maddern GJ (2006). Surgical Simulation. Ann Surg.

[16] Seymour NE, Gallagher AG, Roman SA, O’Brien MK, Andersen DK, Satava RM (2004). Analysis of errors in laparoscopic surgical procedures. Surg Endosc.

[17] Ericsson KA (2004). Deliberate practice and the acquisition and maintenance of expert performance in medicine and related domains. Acad Med.

[18] Zhang X, Lin D, Pforsich H, Lin VW (2020). Physician workforce in the United States of America: forecasting nationwide shortages. Hum Resour Health.

[19] Wunsch C, Buchmann M, Wedel S, Weg PM (2014). Arbeits-und Fachkräftebedarf der Schweiz bis 2060. Abteilung Arbeitsmarktökonomie Wirtschaftswissenschaftliche Fakultät.

[20] Schmidt H, Ostwald D (2022). Fachkräftemangel: Stationärer und ambulanter Bereich bis zum Jahr 2030.

[21] Go MR, Oslock WM, Way DP, Baselice HE, Tamer RM, Kent KC, Williams TE, Satiani B (2020). An updated physician workforce model predicts a shortage of vascular surgeons for the next 20 years. Ann Vasc Surg.

