# Supplementary material for: Virtual reality simulation training in laparoscopic surgery – does it really matter, what simulator to use? Results of a cross-sectional study
Source: BMC Med Educ. 2024 May 28;24:589. doi: 10.1186/s12909-024-05574-0 (PMC11134658; doi:10.1186/s12909-024-05574-0)
Supplement: Supplementary file 1 — Supplementary Material 1. [file 12909_2024_5574_MOESM1_ESM.docx]

| **Exercice Name** | **Simulator** | **Content** | **Virtual Reality*** |
| --- | --- | --- | --- |
| Gallbladder Resection | VM | Candicates perform a complete cholecystectomy from the moment, when Calot’s triangle is exposed. A virtual assistant retracts the gallbladder’s infundibulum. The exercise can be performed as a two-surgeon-procedure with the assistant taking the camera and retracting the gallbladder. | Yes |
| Grasping and bimanual coordination | VM | Three-dimensional numbers must be inserted into recesses. For this purpose, the molds must be handled bimanually and inserted precisely into their target structure. | No |
| Vascular Injury | VM | A bleeding cystic artery stump must be grasped and clipped. | Yes |
| Lap Chole Task 2: Clipping and Cutting with Two Hands | SIM | Candidates must grasp the gallbladder’s infundibulum and clip cystic duct and artery within a designated, highlighted area. | Yes |
| Lap Chole Task 3: Dissection - Achieving a ‘Critical View of Safety’ | SIM | Candidates must dissect the hepatocystic triangle and achieve a Critical View of Safety. | Yes |
| Task 9: Translocation of Objects | SIM | Candidates must bimanually pick up an object and, with a minimum number of of hand-to-hand passes, place the object into a transparent template with the same colored sides. | No |
| PV/T1: Suturing | SS | Candidates must perform a suture and tie a knot on a virtual tissue block | No |
| PV/T2: Lifting & grasping | SS | Candidates must lift a piece of tissue and grasp an object buried underneath, then place the object in a bag. | No |

*Appendix 1: Characteristics of the exercises performed during the VRST training. VM = Virtamed LaparoS^®^, SIM = Simbionix LapMentor III^®^, SS = Surgical Science LapSim^®^.* * "Virtual reality": exercises that simulate real procedures on human tissue
